# P-986. Integration of the National HIV Curriculum (NHC) into health professions programs: faculty and student/trainee reflections and perspectives

**DOI:** 10.1093/ofid/ofae631.1176

**Published:** 2025-01-29

**Authors:** Suyen Schneegans, Trisha Melhado, Eve Asuelime, Nathaniel Teplitskly, Alejandra Garcia-Ponce, Waridibo Allison

**Affiliations:** UNTHSC, Fort Worth, Texas; UNTHSC, Fort Worth, Texas; UNTHSC, Fort Worth, Texas; UNTHSC, Fort Worth, Texas; UNTHSC, Fort Worth, Texas; UNTHSC, Fort Worth, Texas

## Abstract

**Background:**

Targeted Access Knowledge and Education on HIV for Health Professions Programs (TAKE on HIV for Health Professions Programs) is a Health Resources and Services Administration (HRSA) funded effort to support National HIV Curriculum (NHC) integration into health professions programs (HPPs) to cultivate HIV prevention and care knowledge into student/trainee (learners) practice and build the HIV workforce. We report on faculty and learners perspectives within integrating HPPs.

**Methods:**

Using mixed methodology, faculty and learners were asked about their NHC integration experience which was facilitated by the TAKE on HIV training and technical assistance (TTA) Team. Following an integration cycle, faculty participated in 1:1 structured interviews and a focus group to identify facilitators/challenges of integration and sustainment plans. Learners completed a digital survey about time to complete NHC lessons and ratings of HIV knowledge, confidence, and usability of the NHC platform (Likert scale: 1- strongly disagree to 5=strongly agree). Data were analyzed using rapid qualitative analyses, descriptive statistics and mean Likert scores.

**Results:**

Nine participating faculty were from the following HPP disciplines: 3 from Pharmacy, 1 each from Physician Assistant and Family Medicine Residency, and 4 from Nursing. Integration facilitators included TTA support and guidance and NHC student tracking capability. A barrier was learner difficulty with navigating the platform and suggestion for improvement was providing additional visual learner resources for platform navigation. All faculty reported desire to sustain NHC integration with subsequent cohorts and additional NHC lessons. 46 learners completed the survey (13% response rate). 67% reported taking 15-30 minutes to complete an NHC lesson. Overall learners reported having improved HIV knowledge (mean=4.42), confidence in providing HIV care (mean=4.07), and ease of NHC platform navigation (mean=4.29).

**Conclusion:**

NHC integration into HPPs is sustainable if sufficient resources are provided to faculty. TAKE on HIV effectively provides HPP faculty with free TTA resources to facilitate NHC integration and a framework for replicability. HIV knowledge gain amongst learners within integrating HPPs was demonstrated.

**Disclosures:**

**All Authors**: No reported disclosures

